# Through-Scale Numerical Investigation of Microstructure Evolution During the Cooling of Large-Diameter Rings

**DOI:** 10.3390/ma18143237

**Published:** 2025-07-09

**Authors:** Mariusz Wermiński, Mateusz Sitko, Lukasz Madej

**Affiliations:** Department of Applied Computer Science and Modelling, AGH University of Krakow, al. Adama Mickiewicza 30, 30-059 Kraków, Poland; marwer@agh.edu.pl (M.W.); msitko@agh.edu.pl (M.S.)

**Keywords:** phase transformation, cellular automata, cooling, multiscale modelling

## Abstract

The prediction of microstructure evolution during thermal processing plays a crucial role in tailoring the mechanical properties of metallic components. Therefore, this work presents a comprehensive, multiscale modelling approach to simulating phase transformations in large-diameter steel rings during cooling. A finite-element-based thermal model was first used to simulate transient temperature distributions in a large-diameter ring under different cooling conditions, including air and water quenching. These thermal histories were subsequently employed in two complementary phase transformation models of different levels of complexity. The Avrami model provides a first-order approximation of the evolution of phase volume fractions, while a complex full-field cellular automata approach explicitly simulates the nucleation and growth of ferrite grains at the microstructural level, incorporating local kinetics and microstructural heterogeneities. The results highlight the sensitivity of final grain morphology to local cooling rates within the ring and initial austenite grain sizes. Simulations demonstrated the formation of heterogeneous microstructures, particularly pronounced in the ring’s surface region, due to sharp thermal gradients. This approach offers valuable insights for optimising heat treatment conditions to obtain high-quality large-diameter ring products.

## 1. Introduction

The prediction of microstructural evolution during phase transformations is crucial to the development and optimisation of material processing cycles. Phase transformations, driven by thermodynamic and kinetic factors, are responsible for critical properties such as the final product’s stiffness, toughness, and corrosion resistance [1]. Thus, modelling these transformations is important for bridging fundamental material science with industrial applications. Many approaches have been developed to simulate phase transformations, ranging from simple analytical models to advanced computational techniques capable of resolving complex microstructural phenomena across different lengths and time scales [2].

One of the fundamental approaches often used in industry and research is the phenomenological Johnson–Mehl–Avrami–Kolmogorov (JMAK) model, commonly referred to as the Avrami model [3]. It provides a macroscopic description of transformation kinetics under the assumption of random nucleation and isotropic growth [3]. The model’s simplicity allows it to fit experimental data efficiently, and it has been extensively applied to various systems, including recrystallisation, different types of phase transformations, and precipitation simulations [4]. The model is computationally efficient and can even be executed in online control systems. However, despite its advantages, the Avrami model does not account for local microstructural heterogeneities or interactions between growing phases, limiting its predictive capability, especially in non-isothermal conditions. Some of these limitations can be eliminated, e.g., non-isothermal influence [5], but considering microstructure heterogeneities in an explicit manner is still an issue. Therefore, mean-field models were introduced to address the mentioned limitations. These models incorporate average field effects to describe interactions among grains or precipitates, offering a semi-phenomenological bridge between macroscopic kinetics and microstructural features. They are particularly effective for capturing the average behaviour of the investigated material, where the statistical representation of phase distribution is sufficient. Mean-field approaches such as the cluster dynamics model and Kampmann–Wagner numerical (KWN) framework have been successfully applied in modelling, e.g., precipitation kinetics or phase stability in metallic materials [6].

However, these models still neglect local phenomena such as grain boundary pinning, impingement effects, and spatial anisotropies. More complex full-field models have been developed to achieve this [7]. A good example of such full-field approaches is cellular automata (CA) [8] and Monte Carlo (MC) [9] methods, which offer an explicit spatial representation of microstructure. CA models discretise the computational domain into a lattice of regularly distributed CA cells where state transition rules emulate the physical processes governing particular phenomena, e.g., phase transformations [10]. These models are capable of capturing grain growth, solidification, recrystallisation, and texture evolution because they directly consider crystallographic and topological features in the computational rules [11,12]. On the other hand, Monte Carlo simulations are based on local energy minimisation assumptions to model microstructural evolution [13]. Most of the time, the Potts model is used to describe grain boundary energy minimisation. This model class provides detailed results incorporating various local heterogeneities, but the computational time can be excessive. Despite their computational intensity, CA or MC simulations offer high fidelity in modelling thermally activated microstructural changes, making them suitable for materials with complex phase diagrams and kinetics. The paper [14] uses the CA method in combination with the JMAK equation to simulate overlapping phase transformations in high-strength low-alloy (HSLA) steels. The phenomenological approach was used to determine the phase boundary velocity, while the CA method captured the isotropic and anisotropic growth mechanism and the different modes of transformation control for the resulting ferrite and bainite. The authors of [15,16] developed a hybrid model based on frontal cellular automata (FCA) and the lattice Boltzmann method (LBM). Due to the simplicity of implementing the LBM method on graphic processing units (GPUs) and its application to complex geometries, the model enabled efficient calculations of diffusion and heat transfer. Various researchers are also evaluating the execution of these models in high-performance computer systems to minimise the simulation time and provide a research tool for everyday process planning [17].

This study presents a novel multi-scale numerical framework that couples finite element (FE) and cellular automata (CA) methods to simulate the evolution of microstructure during the cooling of large-diameter rings. The model captures location-specific transformation behaviour across the ring thickness and provides practical insights for developing a homogeneous grain size distribution throughout the entire volume of the final product. Therefore, a particular goal of the paper is to capture the evolution of microstructure in large-diameter rings under heat treatment conditions as a guideline for obtaining high-quality products. Two approaches based on simple phenomenological and full-field methods, namely the Avrami and cellular automata models, are used in this case. Designing the heat treatment conditions, especially for large components, is critical for developing processing–structure–property relationships, which are required for the highly demanding and reliable applications.

## 2. Materials and Methods

### 2.1. Cooling of Large-Diameter Rings

A classical analysis of the influence of the ring’s cooling parameters on the microstructure’s development was carried out first. A Ø1376/Ø844 × 508 mm ring hot-rolled from the S355 steel was used for the investigation. The finite element Forge NxT 3.0 program (Transvalor, Biot, France) was used to describe the behaviour of the material at the macroscopic scale during cooling from the fully austenitic regime. Numerical calculations were based on solving the classical thermal problem with radiation and convection:

(1) $$\rho c\frac{\Delta T}{\delta t}=\mathrm{div}\left(k\;\mathrm{grad}\;T\right)$$

where *ρ*—density of the material; *c*—specific heat; *T*—temperature; *t*—time; *k*—thermal conductivity coefficient.

The basic parameters of the thermal model used for the ring material are shown in Table 1.

**Figure 1:**
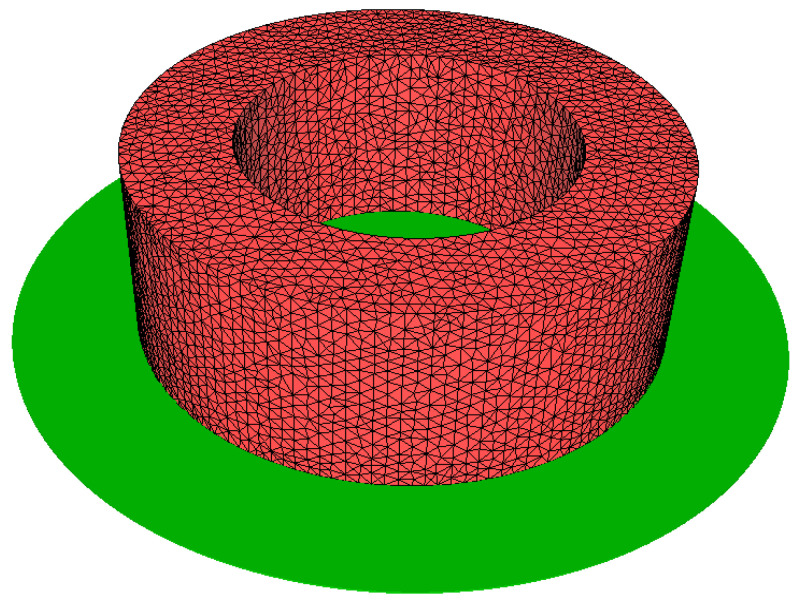
Finite element model for ring cooling simulation.

The large-diameter ring model was discretised with an unstructured mesh of tetrahedral elements, as shown in Figure 1. The initial and boundary conditions are set to replicate the behaviour of the ring during free cooling in air and water quenching. The calculations were performed on a multi-core machine with 32 processors and 64 GB of RAM.

**Figure 2:**
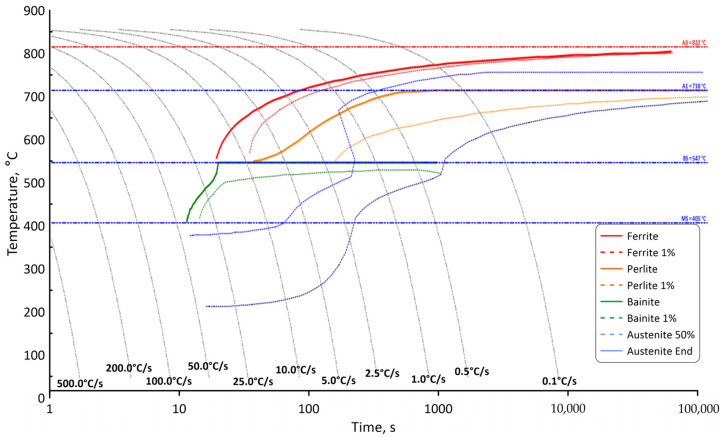
CTT diagram for the investigated steel grade.

At first, the classical phase transformation model based on Avrami’s approach was used during the calculations. For this purpose, information on the continuous cooling transformation (CTT) diagram for the steel grade under consideration (Figure 2) was incorporated into the FE software.

**Figure 3:**
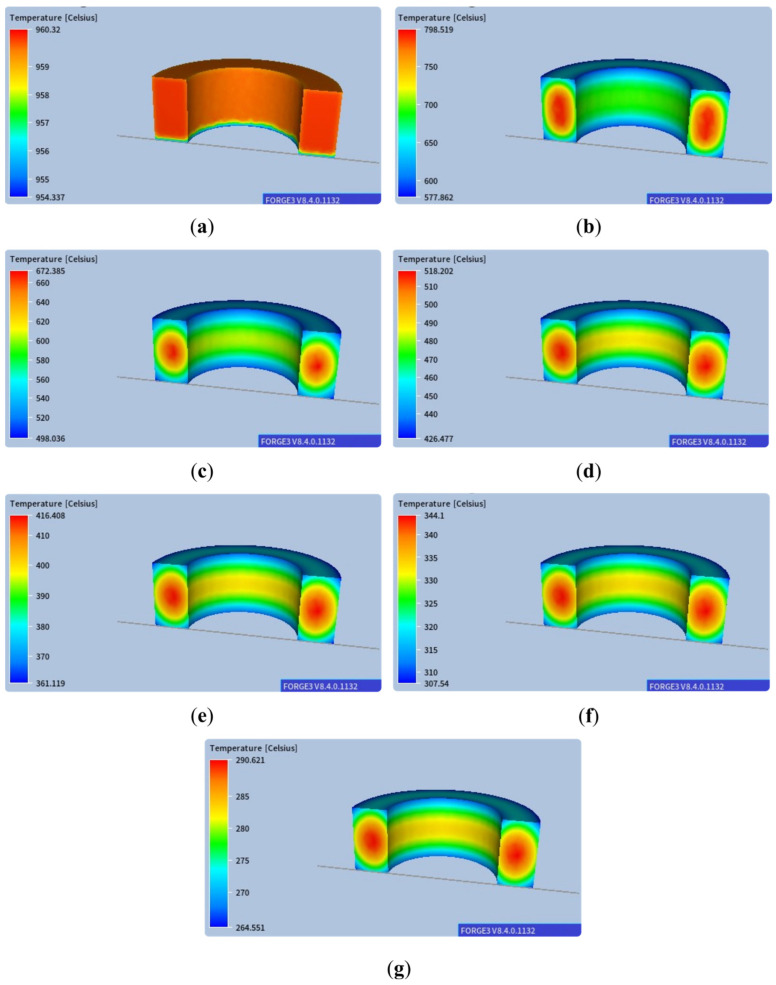
Temperature distributions in successive air cooling stages for a scale in a variable range of values for the following times: (**a**) 0.25 s, (**b**) 2450 s, (**c**) 4950 s, (**d**) 7450 s, (**e**) 9950 s, (**f**) 12,450 s, (**g**) 14,950 s.

An illustration of the temperature distribution during successive stages of air cooling over 15,000 s is shown in Figure 3. In this case, the results of temperature distributions on the ring surface and the cross-section were presented on an adaptive scale so that the temperature gradients could be analysed.

**Figure 4:**
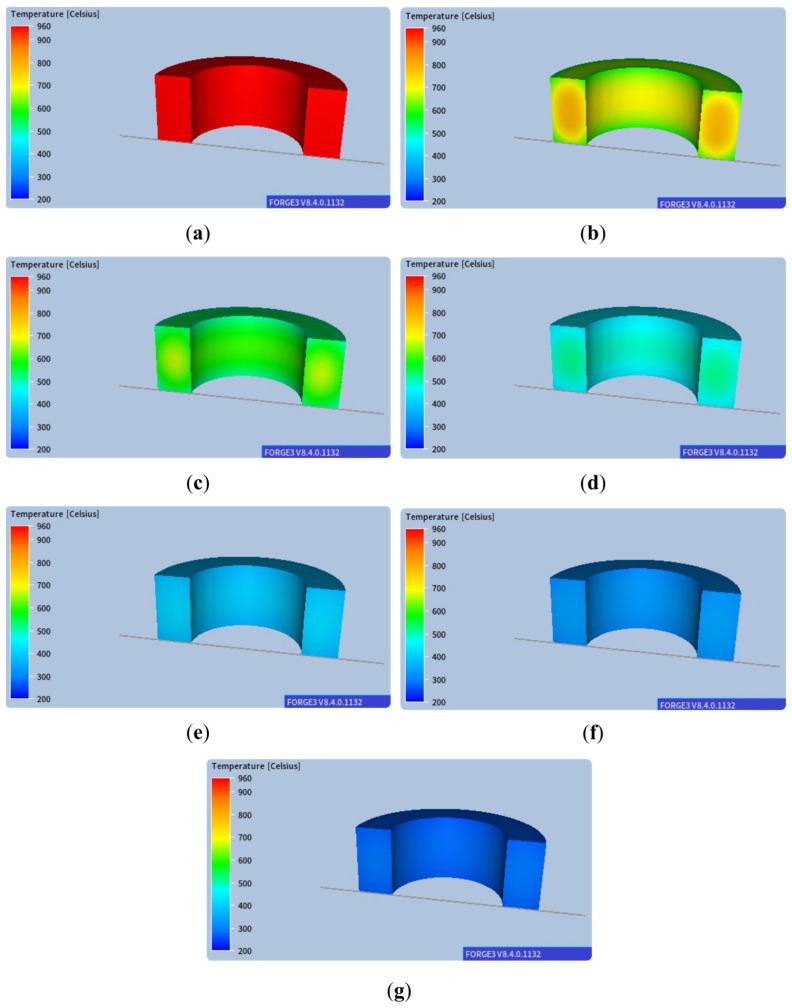
Temperature distributions in successive stages of air cooling for a scale in a fixed range of values for the following times: (**a**) 0.25 s, (**b**) 2450 s, (**c**) 4950 s, (**d**) 7450 s, (**e**) 9950 s, (**f**) 12,450 s, (**g**) 14,950 s.

An analogous temperature distribution is presented in Figure 4, but in this case, the temperature scale is limited by two fixed values for subsequent stages.

**Figure 5:**
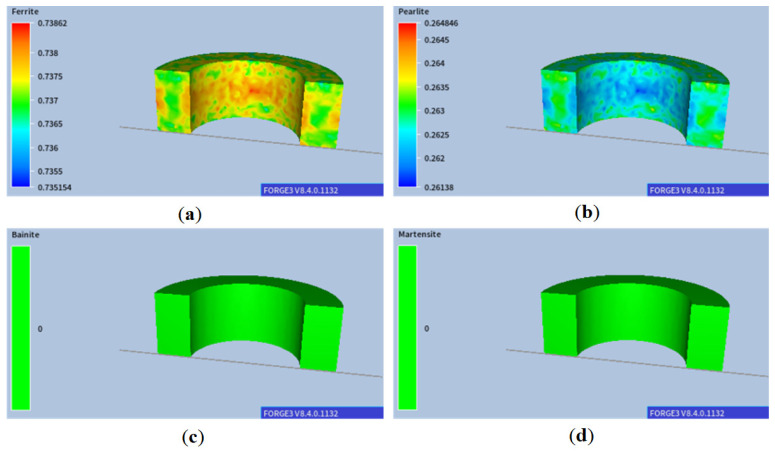
Distributions of the volume fractions of (**a**) ferrite, (**b**) pearlite, (**c**) bainite, and (**d**) martensite after air cooling.

**Figure 6:**
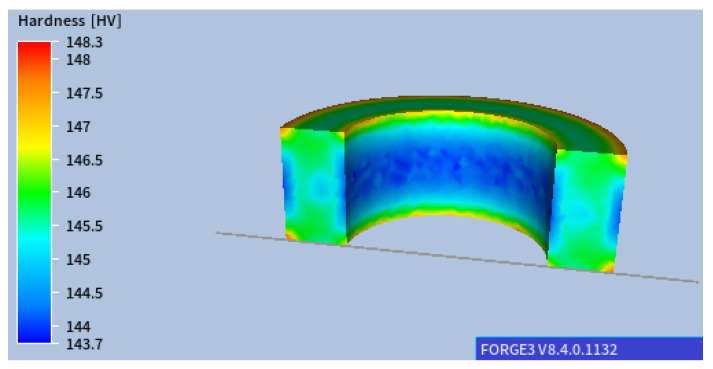
Hardness distributions after air cooling.

As shown, the temperature gradient is visible between the centre of the ring and its edges, and the differences at the edges are particularly significant in the initial cooling stages. With such slow cooling, these differences gradually disappear at lower temperatures. The corresponding results of microstructure development during phase transformation during cooling are presented in Figure 5. As expected, a ferritic–pearlitic structure was obtained with an average ferrite volume of approx. 70%. The results show a slight variation in the volume fraction of individual microstructure components. As a result of such heat treatment, obtaining a homogeneous microstructure of the material with slight differences in hardness is possible, as shown in Figure 6. However, the required hardness for the final component may not be sufficient.

**Figure 7:**
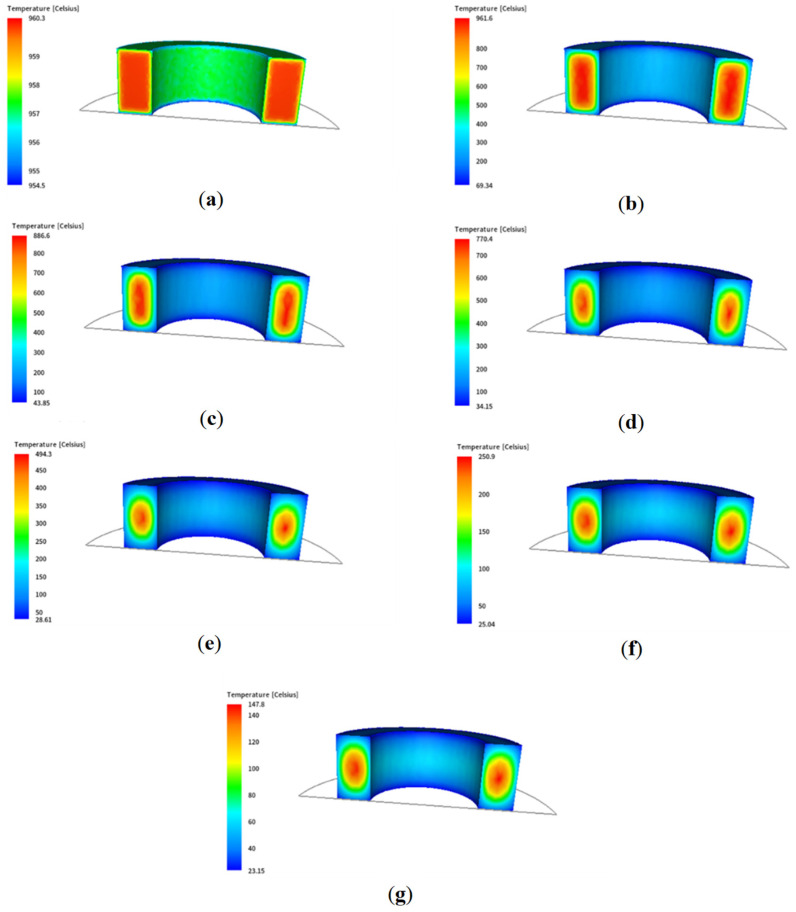
Temperature distributions in successive water cooling stages, for a scale in a variable range of values for the following times: (**a**) 0.25 s, (**b**) 450 s, (**c**) 900 s, (**d**) 1250 s, (**e**) 1550 s, (**f**) 1850 s, (**g**) 2000 s.

Therefore, a second set of calculations was made for the water cooling variant. In this case, as before, it was assumed that the initial temperature of the ring was 960 °C, and the process was carried out until room temperature was reached. An illustration of the subsequent stages of water quenching is shown in Figure 7. Cooling lasted 2000 s, and the temperature gradient between the centre of the ring and its edges is clearly visible.

**Figure 8:**
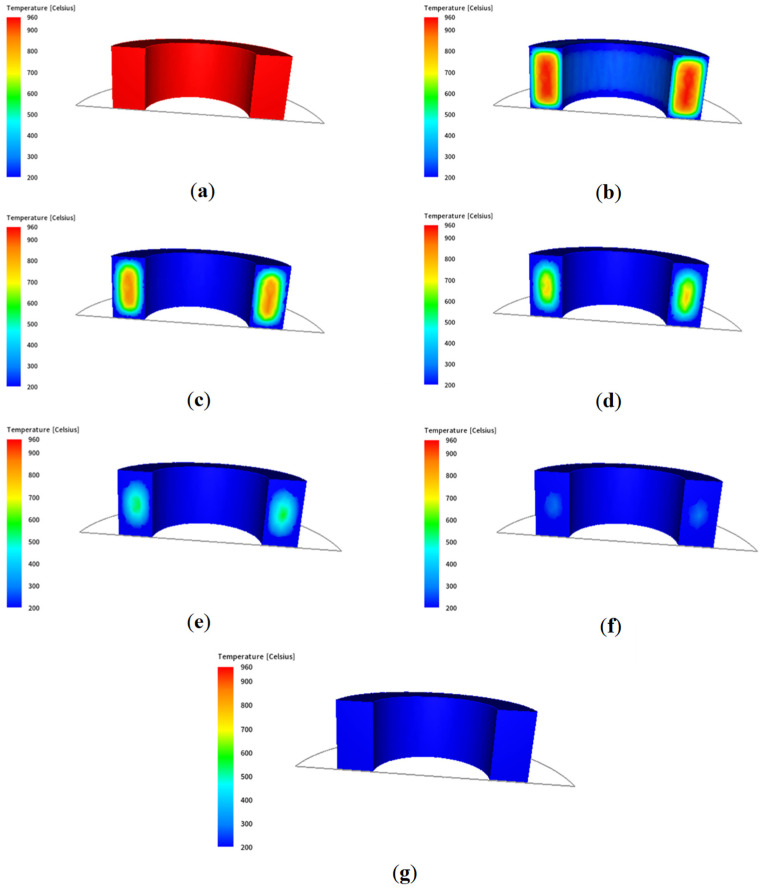
Temperature distributions in successive stages of water cooling, for a scale in a fixed range of values for the following times: (**a**) 0.25 s, (**b**) 450 s, (**c**) 900 s, (**d**) 1250 s, (**e**) 1550 s, (**f**) 1850 s, (**g**) 2000 s.

These differences are visible practically throughout the cooling time up to temperatures below 200 °C (Figure 8).

**Figure 9:**
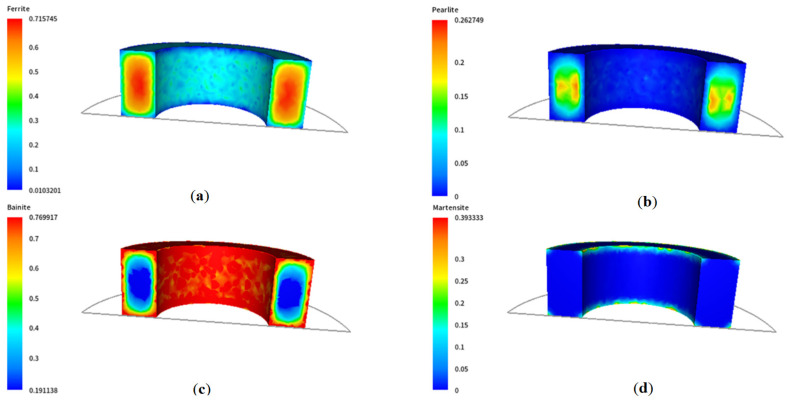
Distributions of the volume fraction of (**a**) ferrite, (**b**) pearlite, (**c**) bainite, and (**d**) martensite after water cooling.

**Figure 10:**
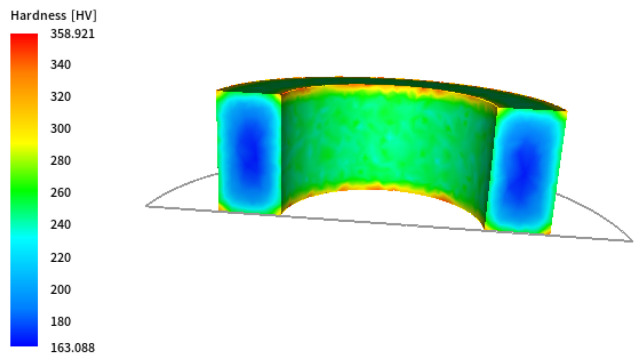
Hardness distributions after water cooling.

The corresponding results of microstructure development during water cooling using Avrami’s model are illustrated in Figure 9. In this case, a microstructure made of ferrite, perlite, and bainite was obtained, which is clearly visible mainly in the near-surface zone. Traces of martensite can also be seen in the material in the area near the edge. As a result of such heat treatment, the finished product is characterised by higher hardness values compared to the case of free cooling, as shown in Figure 10.

The presented results show inhomogeneities in the distribution of individual phase components; however, the obtained information is rather general, and at the microstructure level, it is not possible to observe any heterogeneities. Therefore, as mentioned, such classical analysis was then extended by an investigation with a full-field model developed in-house of phase transformations based on the method of cellular automata.

### 2.2. Cellular Automata Phase Transformation Model

The developed austenite–ferrite phase transformation model is based on the two main algorithms describing the ferrite grains’ nucleation process and subsequent growth. The most important aspects and assumptions regarding the defined internal variables and transition rules describing the progress of the phase transformation are summarised below.

A two-dimensional space of cellular automata with a regular rectangular form and dimensions of *n* × *m* cells was used during the investigation. The number of CA cells and the dimension of a single CA cell are defined as follows: 400 × 400 cells, which corresponds to 200 × 200 µm. During the calculations, the random hexagonal neighbourhood was used:

(2) $$\begin{array}{c} \gamma_{i,j}(t+1)=\left(\begin{array}{ccc} 0 & \gamma_{i-1,j}(t) & \gamma_{i-1,j+1}(t)\\ \gamma_{i,j-1}(t) & \gamma_{i,j}(t) & \gamma_{i,j+1}(t)\\ \gamma_{i+1,j-1}(t) & \gamma_{i+1,j}(t) & 0 \end{array}\right)\\ or\\ \gamma_{i,j}(t+1)=\left(\begin{array}{ccc} \gamma_{i-1,j-1}(t) & \gamma_{i-1,j}(t) & 0\\ \gamma_{i,j-1}(t) & \gamma_{i,j}(t) & \gamma_{i,j+1}(t)\\ 0 & \gamma_{i+1,j}(t) & \gamma_{i+1,j+1}(t) \end{array}\right) \end{array}$$

where *γ_i,j_*—state of the *i*,*j*-th cell.

**Figure 11:**
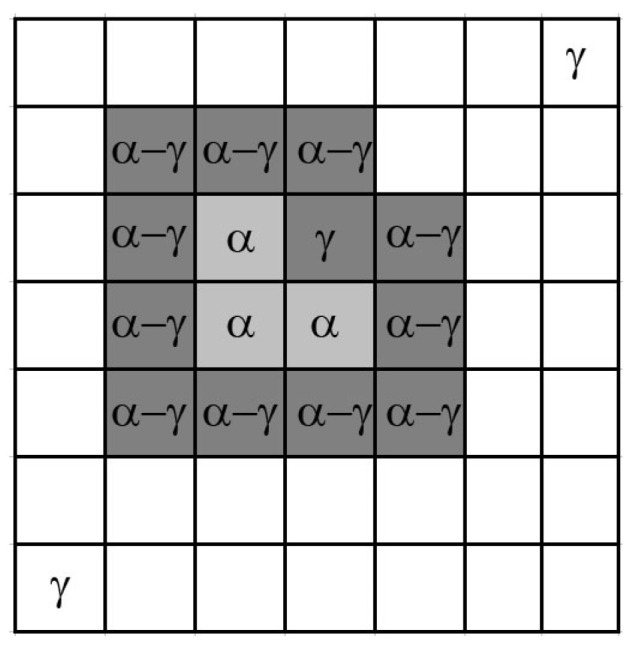
Cells in cellular automata space in the α, γ, and α–γ states.

Three CA cell state variables were defined to reflect the subsequent stages of transformation: *α* –ferrite; *γ*—austenite; α–γ—a CA cell located at the interface between the ferrite and austenite grains (Figure 11).

The nucleation begins when the material is undercooled below the *Ae3* temperature, and the nucleation algorithm is based on the probabilistic approach. The number of nuclei that can be formed in a given calculation step at a given cooling rate is determined by:

(3) $$n^{t}=\frac{a_{1}}{1+\exp\left(\frac{\left(A_{c3}-T_{i}\right)-a_{2}}{a_{3}}\right)}{\left(\frac{B^{t}}{B_{0}}\right)}^{n}p_{nuc}$$

where *a*_1_, *a*_2_, *a*_3_—model parameters (*a*_1_ represents the intensity of possible nucleation density or rate under ideal thermal and mechanical conditions; *a*_2_ represents the threshold or activation offset indicating how large undercooling below *A_c_*_3_ is required to initiate nucleation; *a*_3_ represents a sharpness parameter, which controls the steepness of the exponential response, i.e., how sensitive nucleation is to temperature around the critical point); *A_c_*_3_—temperature of the beginning of transformation; *Ti*—current temperature; *B*_0_, *B_t_*—number of CA cells at the phase interface in the initial and currently analysed time step, respectively; *p_nuc_*—probability.

Depending on the position of the austenite cell, whether it is a triple junction, grain boundary, or grain centre, the probability of nucleation can be modified to take into account differences in, e.g., energy stored as a result of plastic deformation or the density of lattice defects [18]. Changing an austenite cell to a nucleon CA cell (GN) is equivalent to changing the state of the cell from γ to α according to a defined transition rule:

(4) $$Y_{k,l}^{t+1}=\left\{\begin{array}{l} \alpha \Leftrightarrow if\left(l\right)\\ Y_{k,l}^{t} \end{array}\right.l\Rightarrow Y_{k,l}^{t}=\gamma \wedge Y_{i,j}^{t}=\gamma \wedge \mathrm{GN}\left(k,l\right)\neq \mathrm{GN}\left(i,j\right)$$

At the same time, around the nuclei, all adjacent cells change their state to α–γ based on

(5) $$Y_{k,l}^{t+1}=\left\{\begin{array}{l} \alpha /\gamma \Leftrightarrow if\left(l\right)\\ Y_{k,l}^{t} \end{array}\right.l\Rightarrow Y_{k,l}^{t}=\gamma \wedge Y_{i,j}^{t}=\gamma \wedge Y_{i,j}^{t+1}=\alpha$$

The process of ferrite grain growth in the CA model is controlled according to the relation of the grain boundary velocity:

(6) v=MF

The mobility of the grain boundary *M* is described as

(7) $$M=M_{0}\exp\left(\frac{-Q_{t}}{RT}\right)$$

where *M*_0_—coefficient; *Q_t_*—activation energy of the grain boundary diffusion; *R*—gas constant; *T*—temperature.

The driving force *F* of the grain boundary migration process is described by

(8) $$F_{chem}=\mu_{Fe}^{\gamma }-\mu_{Fe}^{\alpha }=\beta \left(C_{eq}-C_{av}^{\gamma }\right)$$

where $\mu_{Fe}^{\gamma }$, $\mu_{Fe}^{\alpha }$—chemical potentials of iron atoms in austenite and ferrite; *β*—model coefficient; *C*—carbon concentrations, equilibrium, and current, respectively.

The current model also provides the possibility to include grain boundary curvature effects using the Kreymayer or Mason models. However, in the current analysis, such effects were not considered due to their negligible impact compared to the dominant chemical driving forces during phase transformation. Similarly, the energy-related driving force from stored deformation energy is not considered, as the transformation occurs after heat treatment, where the stored energy has been vastly relieved. Detailed information on different curvature models available in the presented framework can be found in [19].

According to the above definition, the greater the difference between the equilibrium concentration and the average concentration of carbon in austenite, the greater the driving force for the transformation. During the process, the concentration of carbon in the austenite increases until the transformation is stopped, according to the Fe-C phase equilibrium diagram. Other process-driving forces, e.g., the curvature of the interface between the phases or the energy stored due to preceding deformation, are omitted in the current model.

Therefore, for each CA cell (*i*,*j*) in the state *α*, the grain boundary movement distance *l_ij_* is calculated and used to evaluate the resulting volume fraction *f* in the (*k*,*l*) CA located at the phase boundary in the state α–γ:

(9) $$l_{ij}\left(t\right)=\int_{t_{0}}^{t}vdt$$

(10) $$f_{k,l}=\sum_{1}^{i}\frac{l_{ij}}{L_{CA}}$$

When this fraction exceeds the value of one, the state of the CA cell α–γ changes to α. At the same time, neighbouring cells in the state γ change their state to α–γ. The change in the state of austenite cells by ferrite grain growth presented above is described according to the following formalism of the automata method:

(11) $$Y_{k,l}^{t+1}=\left\{\begin{array}{l} \alpha \Leftrightarrow if\left(l\right)\\ Y_{k,l}^{t} \end{array}\right.l\Rightarrow Y_{k,l}^{t}=\alpha /\gamma \wedge X_{k,l}^{t}>X_{cr}$$

(12) $$Y_{k,l}^{t+1}=\left\{\begin{array}{l} \alpha /\gamma \Leftrightarrow if\left(l\right)\\ Y_{k,l}^{t} \end{array}\right.l\Rightarrow Y_{k,l}^{t}=\gamma \wedge Y_{i,j}^{t}=\alpha /\gamma \wedge Y_{i,j}^{t+1}=\alpha$$

The phase transition model described above has been adapted to simulate structural changes in the S355 steel. For this purpose, the parameters of the CA model were identified based on dilatometric test results and the concept of inverse analysis [20]. The start and end temperatures identified from the dilatometric tests are gathered in Table 2.

**Figure 12:**
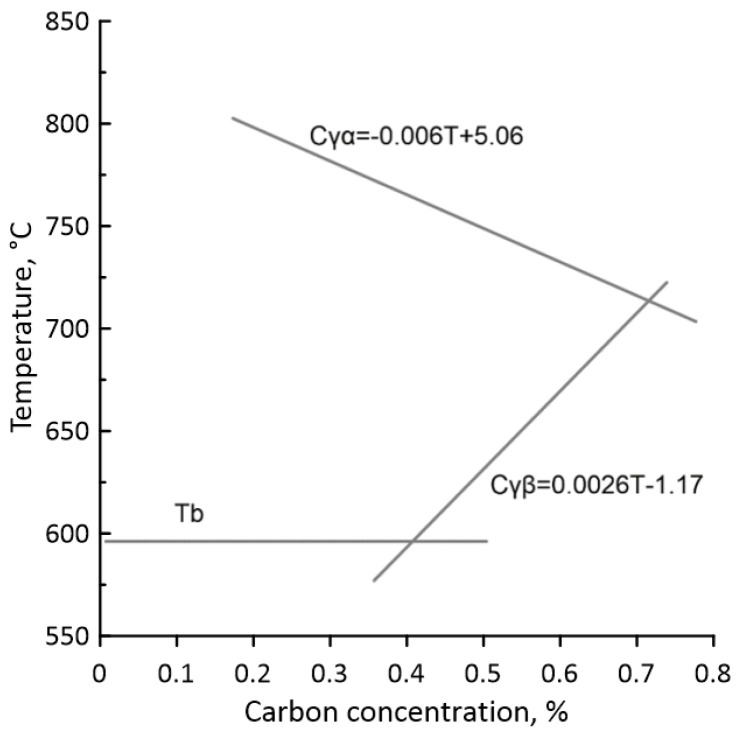
A linearised fragment of the Fe-C diagram defined based on the ThermoCalc calculations (Tb—bainitic transformation start temperature).

Then, the critical temperatures A_c1_ and A_c3_ were identified based on ThermoCalc simulations, and the Fe-C phase diagram in the austenite region was linearised for the CA model’s purposes (Figure 12).

**Figure 13:**
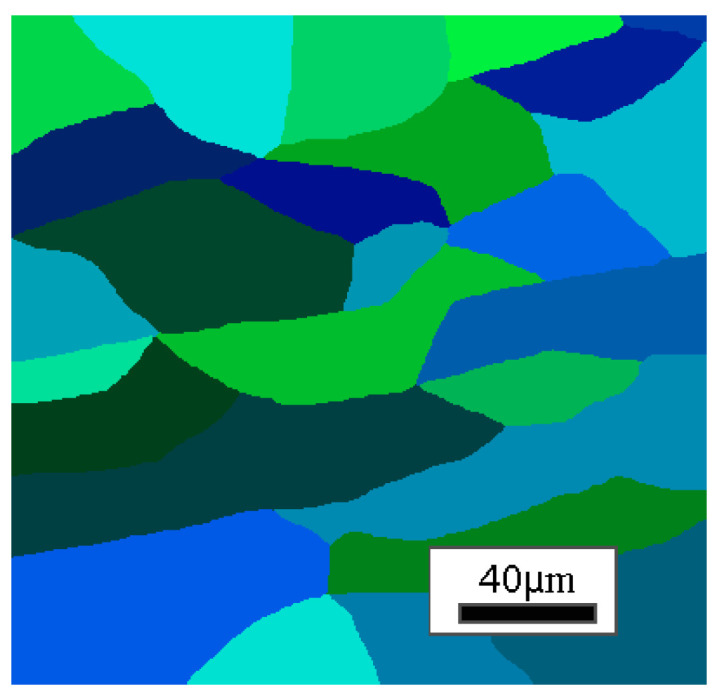
Digital material representation model as input data for inverse analysis (model size 200 × 200 μm).

Then, the direct task model in the inverse analysis algorithm was developed to reflect the selected cooling rates during the dilatometric tests: 1, 2, 4, and 10 °C/s. An input digital material representation model for the CA simulations is shown in Figure 13.

**Figure 14:**
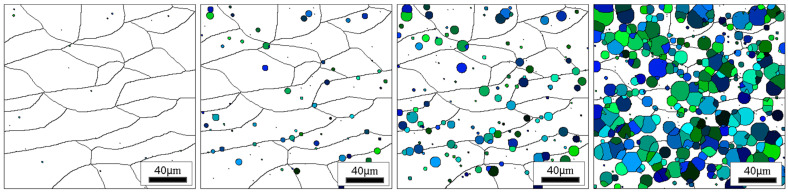
Microstructure morphology during subsequent phase transformation stages. White grains represent the austenite phase (model size 200 × 200 μm, cooling rate 4 °C/s).

Examples of results from the CA simulation revealing the microstructure development during subsequent stages of transformation are presented in Figure 14.

**Figure 15:**
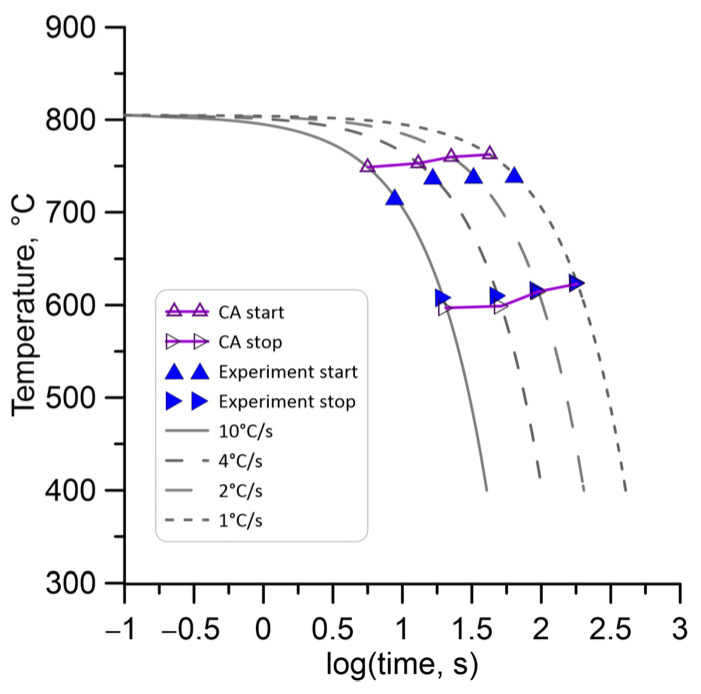
Comparison of the numerically and experimentally evaluated start and end temperatures based on inverse analysis.

The Simplex optimisation algorithm was used to minimise the goal function, which was defined as the mean square root error between the calculated and measured values of the start and end temperature of the transformation for each cooling rate. A comparison of the obtained simulations and measurement results after the end of inverse analysis is shown in Figure 15.

A summary of the identified CA model parameters is presented in Table 3.

**Figure 16:**
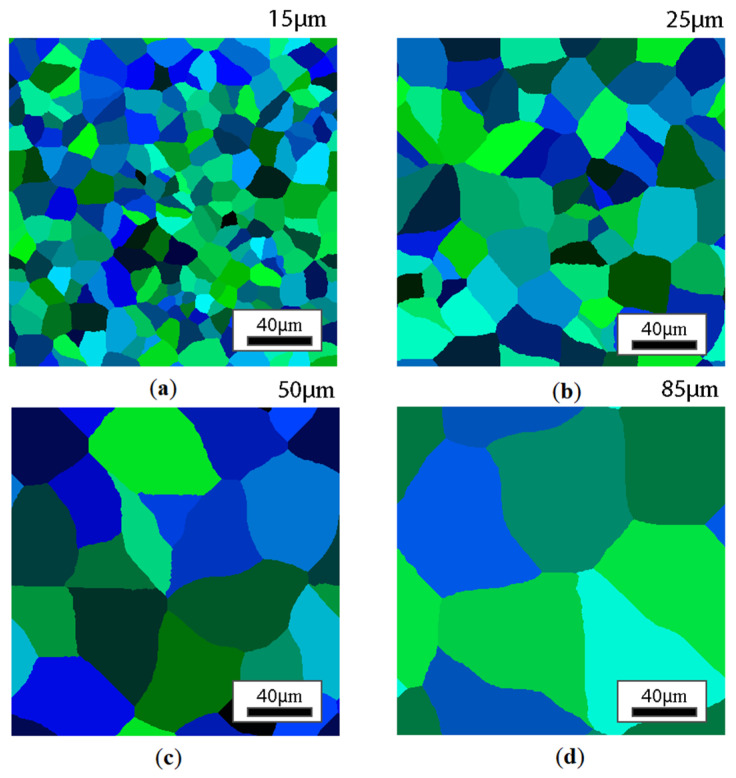
Different variants of the initial microstructure with equiaxial austenite grains with initial grain sizes of (**a**) 15 μm, (**b**) 25 μm, (**c**) 50 μm, (**d**) 85 μm.

**Figure 17:**
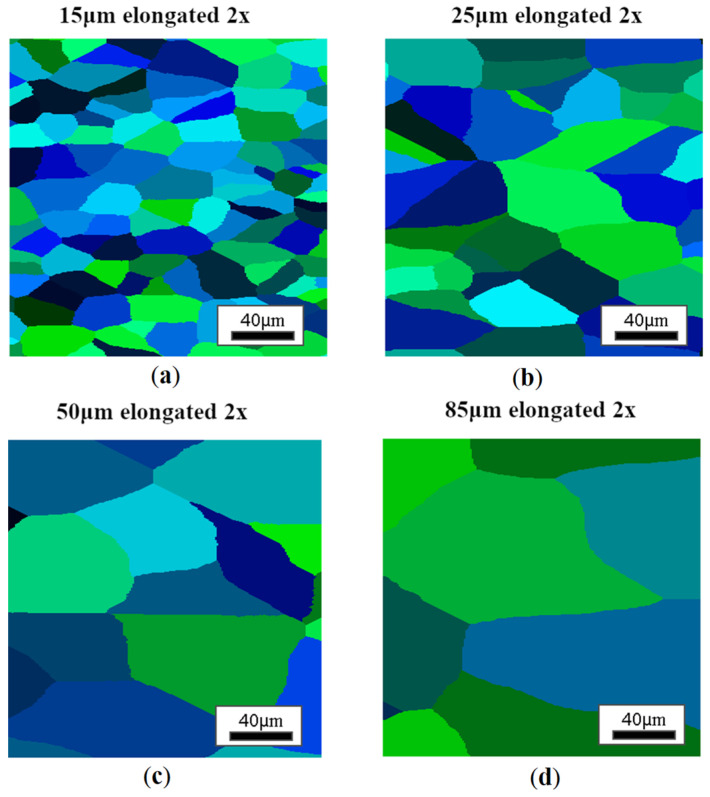
Different variants of the initial microstructure with elongated austenite grains with initial grain sizes of (**a**) 15 μm, (**b**) 25 μm, (**c**) 50 μm, (**d**) 85 μm.

The identified CA model was then used to investigate possible microstructure heterogeneities occurring after the air cooling conditions of a selected microscale model. During this initial analysis, a constant cooling rate was assumed at three levels, 1, 4, and 10 °C/s. In addition, in order to illustrate the role of the initial microstructure on the phase transformation results, four different initial states of the microstructure in the austenite regime were defined with average grain sizes of 85, 50, 25, and 15 μm (Figure 16). In addition, a second set of input data was developed with elongated grains to capture the possible state of grains in the circumferential direction of the ring (Figure 17). Therefore, the two microstructure sets were designed to reflect the state of the microstructure in the longitudinal and transverse directions to the rolling direction. In this case, the unconstrained grain growth model available in the DigiCore library was used to provide an initial digital material representation model.

The two input data sets were used during the phase transformation simulations performed for different cooling conditions according to the parameters summarised in Table 4.

Preparation of an accurate representation of deformed materials for phase transformation simulations can also be performed by using image analysis techniques to process data from metallographic observations. High-resolution microscopy (e.g., electron backscatter diffraction (EBSD) or optical imaging) is often used to capture the initial grain morphology and orientation distribution of the deformed material. These images are then processed through segmentation and reconstruction algorithms to extract grain boundaries, aspect ratios, and orientation data, which are discretized onto the CA grid.

**Figure 18:**
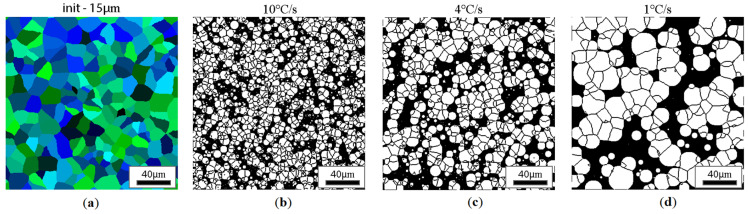
Microstructure obtained for (**a**) an initial grain size of 15 µm and cooling rates (**b**) 10 °C/s, (**c**) 4 °C/s, and (**d**) 1 °C/s. The white grains illustrate the ferrite phase.

**Figure 19:**
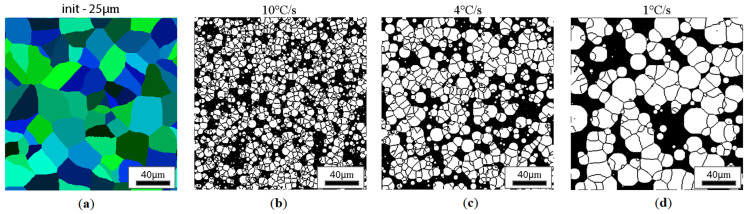
Microstructure obtained for (**a**) an initial grain size of 25 µm, and cooling rates of (**b**) 10 °C/s, (**c**) 4 °C/s, and (**d**) 1 °C/s. The white grains illustrate the ferrite phase.

**Figure 20:**
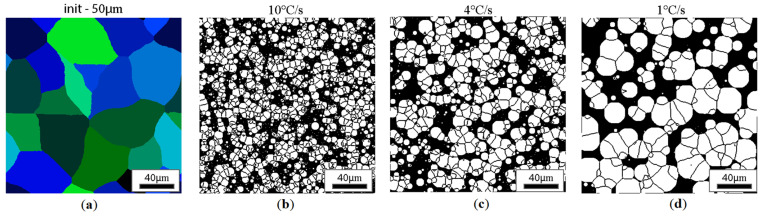
Microstructure obtained for (**a**) an initial grain size of 50 µm, and cooling rates of (**b**) 10 °C/s, (**c**) 4 °C/s, and (**d**) 1 °C/s. The white grains illustrate the ferrite phase.

**Figure 21:**
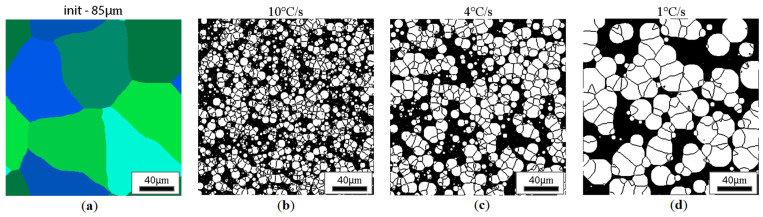
Microstructure obtained for (**a**) an initial grain size of 85 µm, and cooling rates of (**b**) 10 °C/s, (**c**) 4 °C/s, and (**d**) 1 °C/s. The white grains illustrate the ferrite phase.

CA simulation results illustrating the effect of austenite grain size (equiaxial grains) on the morphology of the obtained ferrite grains after subsequent cooling rates are presented in Figure 18, Figure 19, Figure 20 and Figure 21.

At lower cooling rates, large islands of perlite can be found in the final microstructure, while as the speed increases, the dispersion of perlite islands is much greater. Since, during the simulation, there was also a small probability for the appearance of ferrite nuclei inside the austenite grains, both large and small ferrite grains can be observed in all the results, causing a strongly heterogeneous microstructure at the end of the cooling process. If the above-mentioned probability factor were increased in proportion to such a change, the influence of the initial morphology of the microtubule would be reduced. Assuming that the probability of localization of the nucleus is the same everywhere, the material would behave exactly the same as in the classical models.

**Figure 22:**
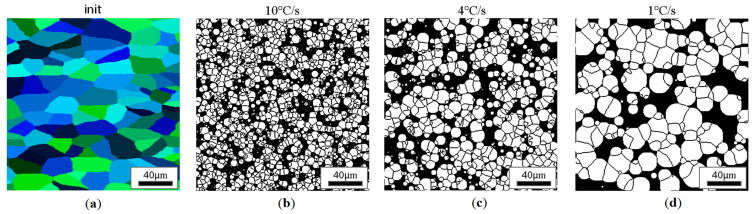
Microstructure obtained for (**a**) an elongated 1:2 austenite grain size of 15 μm and cooling rates of (**b**) 10 °C/s, (**c**) 4 °C/s, (**d**) 1 °C/s. The white grains illustrate the ferrite phase.

**Figure 23:**
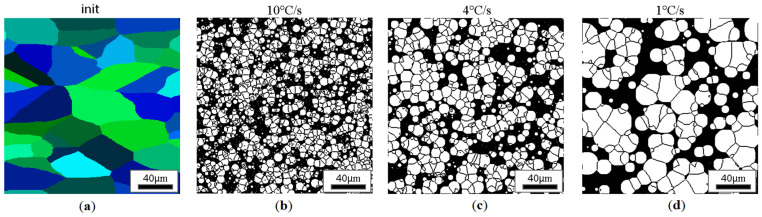
Microstructure obtained for (**a**) an elongated 1:2 austenite grain size of 25 μm and cooling rates of (**b**) 10 °C/s, (**c**) 4 °C/s, (**d**) 1 °C/s. The white grains illustrate the ferrite phase.

**Figure 24:**
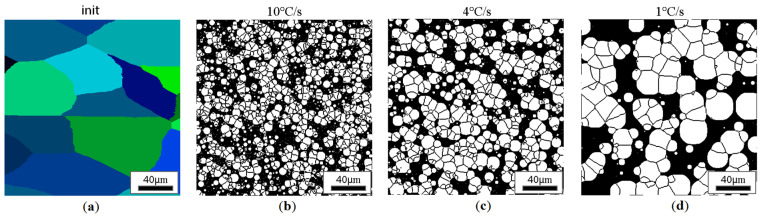
Microstructure obtained for (**a**) an elongated 1:2 austenite grain size of 50 μm, and cooling rates of (**b**) 10 °C/s, (**c**) 4 °C/s, and (**d**) 1 °C/s. The white grains illustrate the ferrite phase.

**Figure 25:**
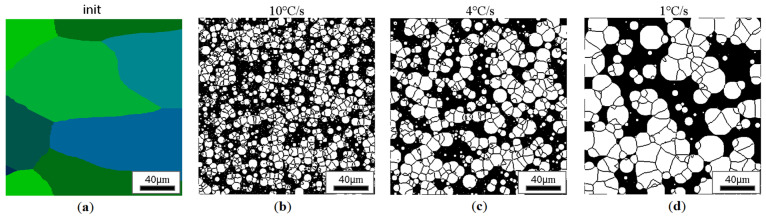
Microstructure obtained for (**a**) an elongated 1:2 austenite grain size of 85 μm, and cooling rates of (**b**) 10 °C/s, (**c**) 4 °C/s, and (**d**) 1 °C/s. The white grains illustrate the ferrite phase.

Similar results for the elongated grains are gathered in Figure 22, Figure 23, Figure 24 and Figure 25.

**Figure 26:**
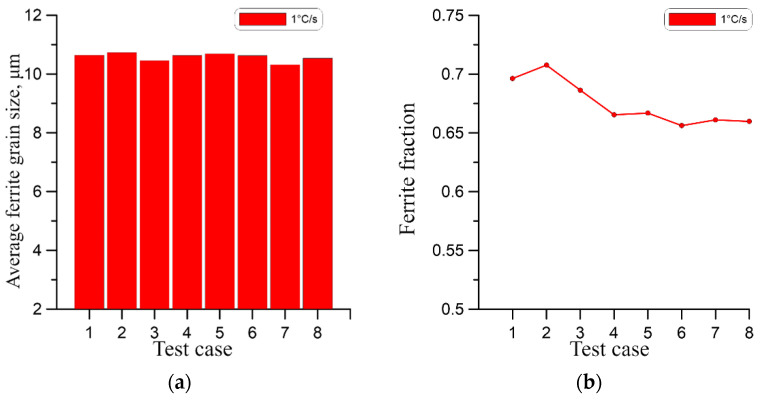
(**a**) Average ferrite grain size, (**b**) ferrite volume fraction for a cooling rate of 1 °C/s.

**Figure 27:**
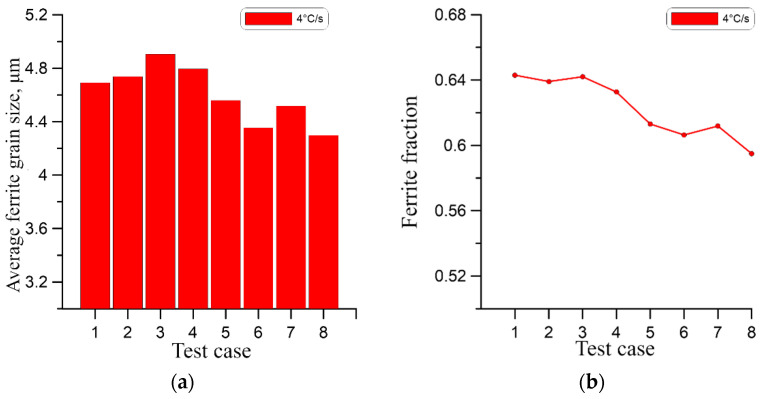
(**a**) Average ferrite grain size, (**b**) ferrite volume fraction for a cooling rate of 4 °C/s.

**Figure 28:**
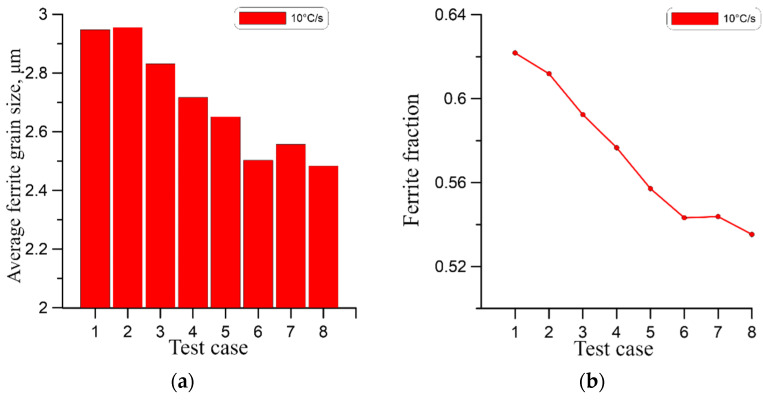
(**a**) Average ferrite grain size, (**b**) ferrite volume fraction for a cooling rate of 10 °C/s.

As presented, the qualitative comparison of the obtained results suggests a very small difference in the final grain size, which may be misleading. Therefore, for a quantitative comparison of the results, the average ferrite grain size at the end of the phase transformation for the cases from Table 3 was calculated as shown in Figure 26a, Figure 27a and Figure 28a. In this case, the effect of grain size for higher cooling rates is apparent.

As can be seen at the highest cooling rate of 10 °C/s, the initial microstructure has the most significant impact on the grain size of the ferrite. At the lower cooling rates of 1 °C/s and 4 °C/s, this effect is progressively less visible. At the same time, in all cases, a high correlation of the ferrite volume fraction with the initial number of austenite grains can be observed in Figure 26b, Figure 27b and Figure 28b. In this case, the ferrite volume fraction decreases with increasing size of the initial austenite grains. However, this relationship is also less pronounced for lower cooling rates.

Finally, such a CA phase transformation model was incorporated into the FE software, and the multiscale model for the simulation of large-diameter rings was established.

## 3. Results from Multiscale Modelling of the Large-Diameter Ring Cooling Process

In the developed model, the data from the FE mesh in the form of temperature profiles obtained after cooling simulations is sent to the CA-phase transformation model to evaluate the final microstructure morphology according to the concept from [21]. A mechanism for importing data from a macroscopic cooling model was implemented to directly transfer the FE results into the CA model. Data from the FE output file is saved to csv (time, temperature) format and then converted to the appropriate list. This data is then linearised within the length of the required time steps. This procedure is performed once at the beginning of the simulation, so there is no need to constantly open and save the FE output text file.

**Figure 29:**
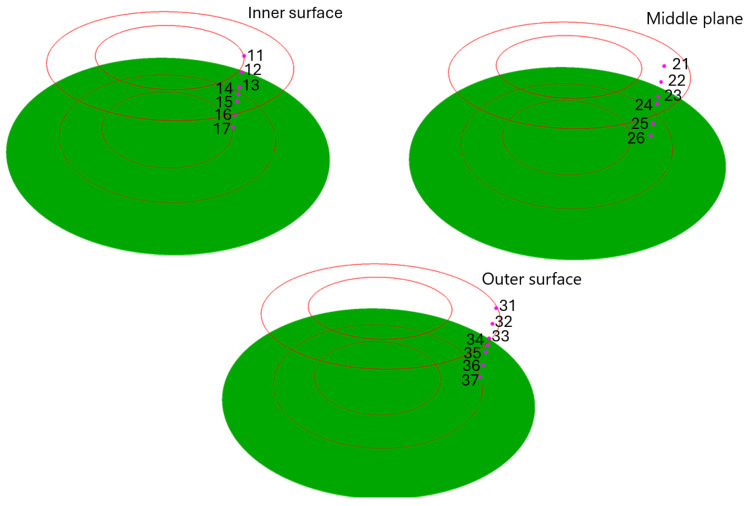
Location of selected points for multiscale calculations with corresponding sensor numbers.

Then, a set of points was defined in the investigated ring, where the cellular automata models are attached. During the research, three sets of measuring points were selected, located on the cross-section of the ring. The points in each set are arranged along the ring’s entire height, as shown in Figure 29.

As mentioned, temperature profiles at characteristic points were extracted from the FE calculations and used as input data for the cellular automata simulations. Again, two different cooling conditions in air and water were used during the investigation.

**Figure 30:**
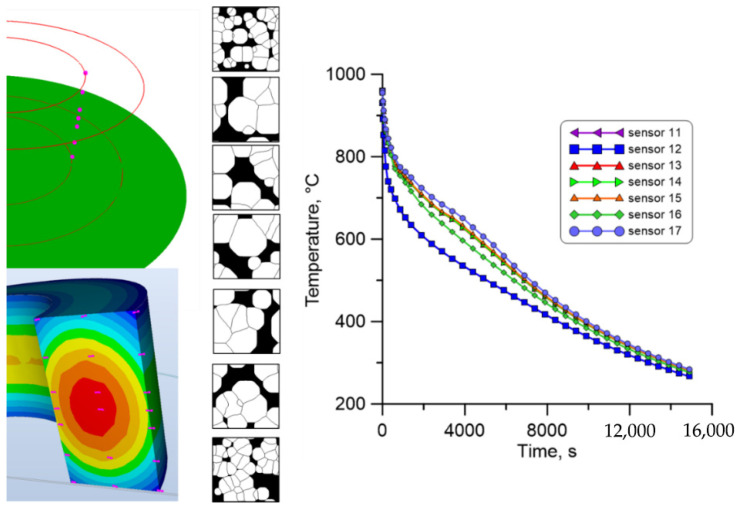
The result from the finite element and cellular automata model for the first set of temperature profiles from the inner surface.

The CA model was finally executed for the 20 investigated measuring points in selected areas of the large-diameter ring, both for free and water cooling. Figure 30 presents the result of CA simulations for the inner part of the ring during free cooling in air. As can be seen at the extreme measuring points, where the cooling rate is the highest, a much larger number of ferrite grains of smaller size are obtained.

**Figure 31:**
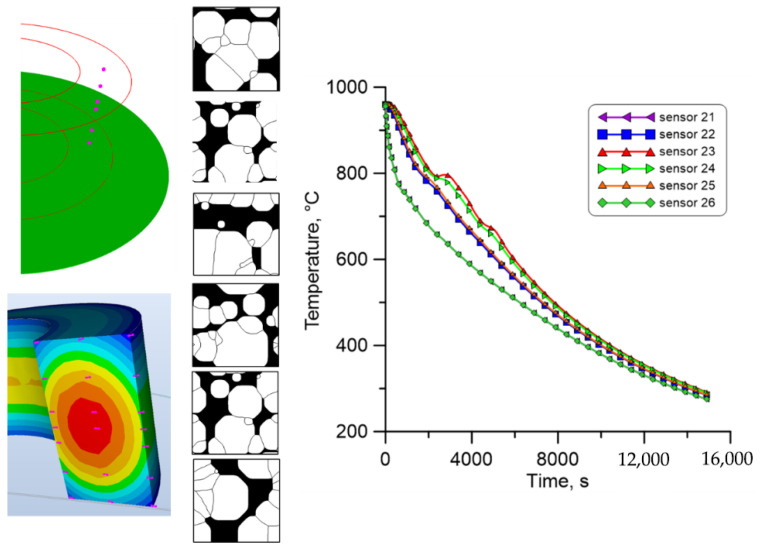
The result from the finite element and cellular automata model for the second set of temperature profiles from the middle plane.

In the second investigated region, i.e., the mid-plane part of the ring (Figure 31), a similar trend can be observed in the number and size of the analysed ferrite grains, which indicates comparable cooling rates in the entire area.

**Figure 32:**
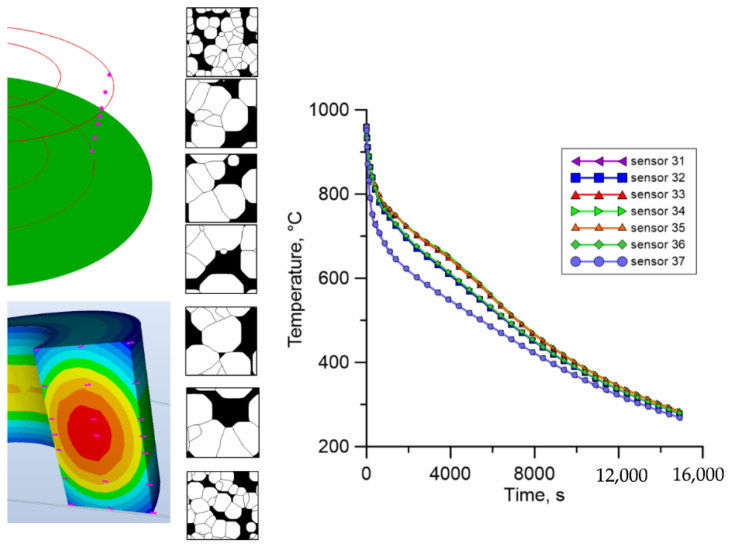
The result from the finite element and cellular automata model for the third set of temperature profiles from the outer surface.

Finally, the results for the outer part of the ring (Figure 32) are similar to the results obtained for the internal part. Additionally, a much larger number of new ferrite grains can be observed for the most extreme points.

**Figure 33:**
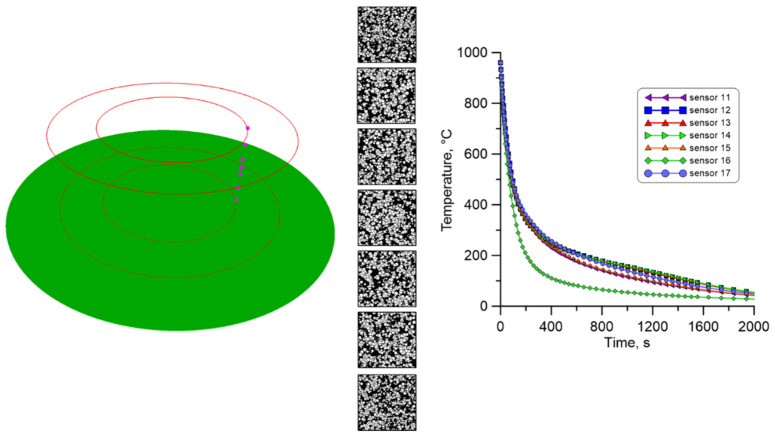
The result from the finite element and cellular automata model for the first set of temperature profiles.

The same investigation was conducted for the ring during water cooling. Figure 33 shows the result of the calculation of changes in microstructure for the inner part of the ring. As can be seen at the extreme measuring points, where the cooling speed is significant, a higher number of ferrite grains was obtained, but these differences are negligible compared to cooling in air. It should be noted, however, that in the obtained results, outside the ferrite phase, the dark regions of the digital microstructure represent a conglomerate of perlite and bainite.

**Figure 34:**
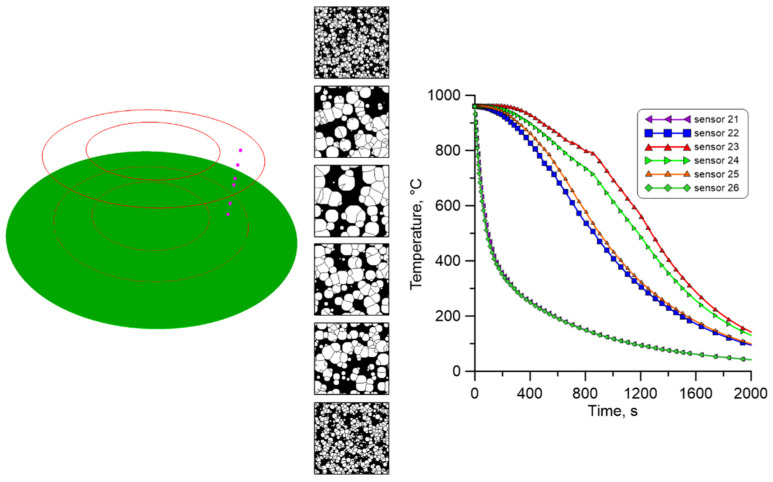
The result from the finite element and cellular automata model for the second set of temperature profiles.

In the central part of the ring (Figure 34), a similar trend can be observed in the number and size of ferrite grains at internal and extreme measurement points, which also indicates differences in the cooling speed of near-surface layers. The central areas are characterised by a much larger average grain size than the microstructure located near the surface.

**Figure 35:**
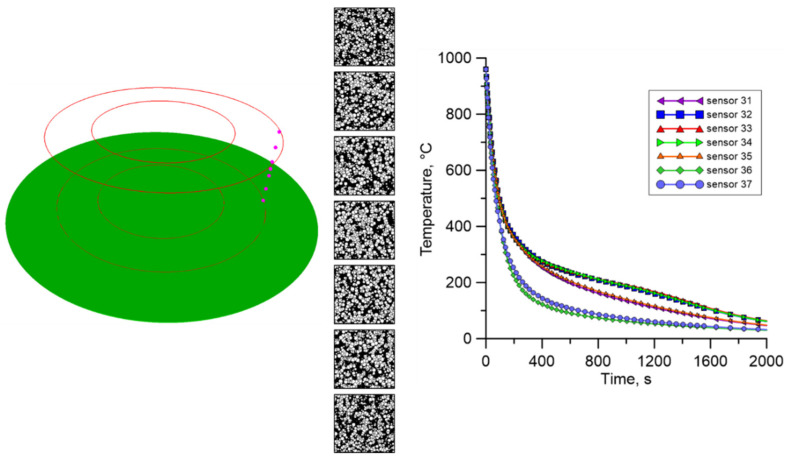
The result from the finite element and cellular automata model for the third set of temperature profiles.

Finally, the results for the outer part of the ring (Figure 35) are generally the same as the results obtained for the internal part. Additionally, a greater number of new ferrite grains can be observed for the most extreme points, but these differences are not significant. The similarity in microstructure evolution between the inner and outer sections of the ring can be explained by the thermal boundary conditions and the symmetry of ring geometry. Additionally, heat conduction within the ring cross-section redistributes thermal energy, contributing to similar cooling rates near the outer and inner surfaces after an initial transient period. As a result, the temperature evolution and phase transformation kinetics at the inner and outer surfaces become aligned, resulting in consistent microstructural features in these regions.

The results indicate a clear fragmentation of the ferrite grain in the near-surface area, which affects the heterogeneity of the average grain size on the cross-section.

The presented results indicate that water cooling promotes a more rapid phase transformation and finer grain structures at the outer surface but also introduces elevated thermal gradients that may contribute to the development of residual stress. In contrast, air cooling provides a slower and more uniform cooling profile, resulting in coarser grains but improved temperature homogeneity. These observations suggest that a combined cooling strategy involving initial rapid cooling followed by slower air cooling may offer a balance between microstructural refinement and thermal stability. The findings offer general insight into the phase transformation behaviour during ring cooling, while the industry should develop specific process strategies tailored to individual part geometries, material systems, and performance requirements.

As presented, the developed multi-scale simulation setup provides information on the volume fraction of individual components of the microstructure and local inhomogeneities in the size of grains. This information can be directly used in the design of appropriate cooling conditions for such large-scale components.

## 4. Discussion

Three different analyses with varying levels of complexity were conducted within the current research: the first with the classical FE model coupled with the Avrami-type microstructure evolution approach, the second with the full-field cellular automata microstructure evolution model, and finally, the coupled FE and CA in a multiscale approach. All these approaches were applied to the S355 large-diameter steel ring to evaluate microstructural evolution during cooling after the hot ring rolling operation. Two cooling approaches were examined, air cooling and water quenching, resulting in distinct thermal gradients and microstructure outcomes. In terms of air cooling, after approx. 15 000 s, a mostly ferrite–pearlite structure (~70% ferrite) was obtained, which is characterised by quite low hardness. On the other hand, after water quenching, which was around 7× faster, a ferrite–pearlite–bainite structure, with some localised martensite formation near the ring edges due to steeper thermal gradients, was obtained. Such a microstructure will be less uniform but provide much higher hardness values. The investigation based on the Avrami-type model provided general information about phase transformation. Despite capturing temperature distributions and general microstructural outcomes, such an approach could not provide information on microstructural heterogeneities. However, as presented, the developed full-field 2D CA model to simulate austenite-to-ferrite transformation can successfully address this issue. The full-field model can incorporate nucleation and grain growth mechanisms under varying cooling conditions. Temperature, chemical potential differences, and carbon concentration gradients can then be considered to predict grain growth. The inverse analyses and dilatometric tests are essential to determine the model parameters. However, in [22], the authors presented a promising approach to the exploration and determination of model parameters based on Bayesian optimization that could be used as an alternative. The outcome from CA simulations showed that large perlite islands and heterogeneous ferrite grain sizes are present at low cooling rates. At the same time, finer ferrite grains and a more dispersed microstructure are obtained after higher cooling rates. It was also pointed out that the initial austenite grain size and grain shape (equiaxed vs. elongated) significantly influence the final ferrite grain size and volume fraction. This phenomenon is especially pronounced at higher cooling rates like 10 °C/s. The accuracy of the solution depends on several factors, firstly, how many measurement points and correlating microstructures will be used in the multi-scale model, and additionally, how often the temperature change history data will be recorded and on what basis the initial data will be prepared in the form of microstructure after deformation—in this case after ring rolling—into the CA model. The model reduction domain would be an interesting subject of further research.

Finally, coupling the FE model with CA simulations additionally increases the model’s predictive capabilities, as presented in multiple literature studies [23,24]. To make the process computationally efficient, output files with time–temperature data from FE simulations have to be exported and interpolated for CA input in an automatic manner. While the primary focus of this work is on the predictive capabilities of the multi-scale model, the importance of computational performance, particularly for industrial-scale applications, is also emphasized. For the phase transformation (PT) model, maintaining numerical stability during the diffusion step is critical and can be achieved using fixed or adaptive time stepping. For example, simulating a 300 × 300 cell microstructure with a cooling rate of 1 °C/s required approximately 6400 s with a fixed step, compared to 1000 s with adaptive stepping [25]. Since 24 spatial points were analysed sequentially, this results in a significant computational demand. Although optimization through parallel computing is beyond the scope of the current study, ongoing work is addressing parallelization strategies for CA-based models [26].

Such a hybrid model provides enhanced insight into spatial microstructure heterogeneities and enables local property prediction, which is essential for further optimising thermal treatment in ring production. This can be confirmed by the work [27], where the authors present a similar model for hot-rolled steels, also based on the CA and FE methods. They used the FE approach to supplement the temperature and strain energy information to the CA model, which ultimately allowed for the simulation of various types of recrystallisation and the austenite-to-ferrite phase transformation in advanced high-strength steel (AHSS), taking into account the solute drag effect and accumulated strain energy. This model allows the accurate prediction of the fraction and average grain size of ferrite. This means that models of this type perform well in simulating complex phenomena with the consideration of different scales.

## 5. Conclusions

This study demonstrates the effectiveness of a multiscale modelling approach for predicting microstructure evolution in large-diameter steel rings during thermal processing. By integrating finite element thermal simulations with both the Avrami phase transformation model and a detailed cellular automata framework, it is possible to capture the interplay between macroscopic heat flow and microscopic grain evolution. The results revealed that ring geometry and cooling method significantly influence thermal gradients, leading to heterogeneities in the resulting microstructure. Air cooling produced relatively uniform microstructures with gradual gradients, while water quenching resulted in pronounced differences between surface and core regions, with finer ferrite grains and higher hardness localised near the surface. The Avrami model offered valuable insight into overall transformation kinetics but lacked spatial resolution, which was effectively addressed using the cellular automata model. The latter allowed detailed simulation of grain nucleation and growth processes, showing how initial austenite grain size and morphology variations substantially affect the final grain structure. Specifically, finer or elongated grains enhanced nucleation rates and promoted ferrite refinement, especially at higher cooling rates. These findings underscore the importance of initial microstructure and processing parameters in determining final material properties. Overall, the proposed framework provides a robust framework for simulating microstructural evolution under industrially relevant cooling conditions and offers a pathway to the more precise control of mechanical properties through process optimisation.

Future research will focus on explicitly including pearlite, bainite, and martensite formation during the CA simulations. The second direction will be related to the transition from the 2D to 3D CA model to capture grain morphology more accurately, especially for elongated or non-uniform grain shapes.

## Figures and Tables

**Table 1:** Thermal model parameters.

Specific Heat, $\frac{J}{kg\;K}$	Density, $\frac{kg}{m^{3}}$	Conductivity Coefficient $\frac{W}{m\;K}$	Emissivity Coefficient $\frac{W}{m^{2}}$
778	7850	35.5	0.88

**Table 2:** Start and end temperatures identified from the dilatometric tests.

Q, °C/s	Ferrite Start log(t), s	Temp, °C/s	Q, °C/s	Ferrite Stop log(t), s	Temp, °C/s
1	1.806	741	1	2.257	2.257
2	1.511	740	2	1.975	1.975
4	1.217	739	4	1.687	1.687
10	0.944	717	10	1.294	1.294

**Table 3:** Identified parameters of the austenite–ferrite phase transformation model.

*Q* [kJ/mol]	*a*_1_	*a*_2_	*a*_3_	*M*_0_	*β*
139.2	980	280	−50	4.00 × 10^−6^	6.00 × 10^−6^

**Table 4:** Simulation variants.

Test Case No.	Initial Grain Size	Analysed Cooling Rate
1	15 μm	1, 4, 10 °C/s
2	15 μm elongated 2×	1, 4, 10 °C/s
3	25 μm	1, 4, 10 °C/s
4	25 μm elongated 2×	1, 4, 10 °C/s
5	50 μm	1, 4, 10 °C/s
6	50 μm elongated 2×	1, 4, 10 °C/s
7	85 μm	1, 4, 10 °C/s
8	85 μm elongated 2×	1, 4, 10 °C/s

## Data Availability

The original contributions presented in this study are included in the article. Further inquiries can be directed to the corresponding author.

## Abbreviations

The following abbreviations are used in this manuscript:

JMAK	Johnson–Mehl–Avrami–Kolmogorov
CA	Cellular Automata
MC	Monte Carlo
KWN	Kampmann–Wagner numerical framework
FE	Finite Element
HSLA	High-strength low-alloy
AHSS	Advanced high-strength steel
LBM	Lattice Boltzmann method
GPU	Graphical processing unit
EBSD	Electron Backscatter Diffraction
DMR	Digital Material Representation
CTT	Continuous Cooling Transformation
